# “Healthcare worker perceptions of healthcare-associated infections and health inequity”

**DOI:** 10.1017/ash.2023.196

**Published:** 2023-08-03

**Authors:** Laura L. Pedersen, Rachel Pryor, Gonzalo Bearman

**Affiliations:** 1 Virginia Commonwealth University, Richmond, VA, USA; 2 Virginia Commonwealth University Health System, Richmond, VA, USA

Healthcare-associated infections (HAIs) in the acute care setting occur after a patient is admitted for 48 hours or longer. These infections include central line-associated bloodstream infections (BSIs), catheter-associated urinary tract infections (CAUTIs), surgical site infections, ventilator-associated events, and clostridium difficile infections (CDIs).^1,2^ Studies estimate that 4% of patients admitted to acute care hospitals will experience an HAI.^3^ This burden in the United States amounts to 648,000 patients with 721,800 infections a year.^3^ There is novel data suggesting that patients of color experience higher rates of HAIs compared to White inpatients.^4,5^

The association of race and ethnicity to specific infections and outcomes is published.^1,4–7^ Researchers reported an increased BSI risk in non-Hispanic African Americans when compared to non-Hispanic Whites (hazard ratio 1.31, 1.02–1.69).^6^ White patients experience higher incidence of CDI than African American patients, though African American patients with CDI experience higher mortality, more cases of severe CDI, and longer lengths of stay.^1^ Asian American and Hispanic patients have higher rates of CAUTI than Caucasian patients.^4^African Americans have higher rates of hospital-acquired methicillin-resistant *Staphylococcus aureus* (MRSA) infections.^7^

In addition to race and ethnicity as influencers of HAI incidence and outcomes, English as a second language also impacts inpatient outcomes. Limited English proficiency (LEP) increases risk for hospital readmission and increases risk of mortality in patients with sepsis.^8^

We evaluated healthcare workers (HCWs) knowledge and perceptions of racial and ethnic inequities related to HAIs and hospital outcomes.

## Methods

We utilized a voluntary, anonymous survey with 8 Likert scale questions. Survey questions reflected published data identifying racial and ethnic disparities in HAIs. The Likert scale offered 5 responses, including 1 neutral response. After institutional board review approval, electronic surveys in REDCap form were distributed via e-mail at an academic medical center. REDCap is a data management system with the ability to collect, store, and export responses to analysis software. Surveys were distributed to care partners, respiratory therapists, radiology technicians, mid-level providers, nurses, medical doctors, nursing students, and medical students. E-mail address pools were provided by the respective administrative staff. Only HCWs in adult surgical and medical intensive care units and general units were included. No demographic data such as age, race, or ethnicity were recorded. The surveys were distributed four times in 1-week intervals between August and October 2022.

## Results

Respondents completed 370 surveys for a response rate of 12.2%. Self-identified roles included medical doctor (MD) (106), registered nurse (RN) (98), mid-level provider (61), care partner (15), MD student (25), radiology technician (5), respiratory therapist (16), RN student (17), and other/did not indicate (27).

Aggregated survey responses are presented in Table 1. In terms of LEP, 87% of respondents agreed that LEP increases risk for hospital readmission. Approximately 66% of respondents agreed that LEP increases risk of mortality in patients with sepsis.

**Table 1.** Survey responses

Question	Cumulative responses		
	Agree	Disagree	Unsure
1. Limited English proficiency (LEP) increases risk for hospital readmission.	87% (322/370)	4% (13/370)	9% (35/370)
2. LEP increases risk of mortality in patients with sepsis.	66% (243/370)	6% (23/370)	28% (104/370)
3. African Americans have higher rates of hospital-acquired MRSA infections.	25% (94/370)	9% (32/370)	66% (244/370)
4. Caucasian people have higher rates of Clostridium difficile (C diff) infections than African Americans.	12% (45/370)	12% (46/370)	75% (279/370)
5. Compared to Caucasian patients, African Americans with C diff infections have a higher risk for more severe infections.	37% (138/370)	7% (26/370)	56% (206/370)
6. Compared to Caucasian patients, African Americans with C diff infections have a higher risk for mortality.	42% (155/370)	7% (27/370)	51% (188/370)
7. Asian American and Hispanic patients have higher rates of catheter-associated urinary tract infections than Caucasian patients.	23% (84/370)	10% (37/370)	67% (249/370)
8. African American patients have a higher risk of hospital-acquired bloodstream infections than Caucasian patients	25% (94/370)	9% (32/370)	66% (244/370)

In terms of HAIs, most respondents were unsure of racial and ethnic disparities affecting patient outcomes. Most (65.9%) were not sure if African Americans have higher rates of hospital-acquired MRSA infection and only 25.4% agreed. Most respondents (65.9%) were unsure if African Americans have a higher risk of hospital-acquired BSI than Caucasian patients. Two-thirds of HCWs were unsure if Asian and Hispanic patients have higher rates of CAUTI compared to Caucasian patients.

Only 12.1% of respondents agreed that Caucasian patients have higher rates of CDI than African American inpatients. Approximately half of respondents were unsure if African Americans have a higher risk of severe CDI and related mortality, 55.7% and 50.8%, respectively.

## Discussion

Most HCWs were not aware of the influence of race and ethnicity in the prevalence of HAIs and associated outcomes. Only 25.4% of participants agreed and 65.9% were unsure that African American patients have increased risk of MRSA infections compared to White patients. Nevertheless, African American patients have a relative risk of 3.22 (95% confidence interval [CI], 2.35–4.35) for hospital-onset MRSA infection.^7^ African American and Hispanic hemodialysis patients are at higher risk for *Staphylococcus* BSIs.^10^ Similarly, 66.7% of HCWs were unsure if Asian American and Hispanic patients have higher rates of CAUTI compared to Caucasian patients. This disparity has been previously described in the literature and is potentially related to language barriers.^4^

The survey revealed an overall lack of awareness of racial disparities in CDI. Only 12.1% HCWs agreed that Caucasian people have higher rates of CDI, nevertheless Caucasian patients make up 90% of cases.^1^ Most HCWs surveyed were unsure if African Americans have high risk for severe CDI and death. Caucasian patients make up overwhelming most CDI, but African American patients have a higher risk of both mortality and severe CDI.^1^

We are the first to report HCW perceptions of the impact of health inequity on HAI risk and outcomes. At the individual HCW level, we observed a lack of provider awareness of HAI risks in minority groups. HCWs not being aware of increased risk for HAIs in minority groups compared to Caucasian patients may perpetuate health inequity and worsen patient safety outcomes.

The primary study limitation is a low response rate. Each recipient had the opportunity to complete the survey over a 4-week period. We do not believe that additional attempts beyond four cycles of survey distribution will increase response rate significantly. Additionally, this is a single academic center study design. These data may not be generalized. Study strengths include using a voluntary, anonymous survey tool, and a diverse distribution of HCW roles.

Nonwhite patients experience higher rates of HAIs. This includes CDI, hospital-acquired BSI, and CAUTI. We report a lack of HAI racial disparities awareness by HCWs in a single-center survey. This study is the first to our knowledge to assess HCWs’ knowledge of health inequity and HAIs. Future studies are needed to better understand this trend and to define strategies for heightened awareness of HAI inequities.

## References

[1] Argamany JR , Delgado A , Reveles KR. Clostridium difficile infection health disparities by race among hospitalized adults in the United States, 2001 to 2010. BMC Infect Dis. 2016;16:454. 27568176 10.1186/s12879-016-1788-4 PMC5002147

[2] Monegro AF , Muppidi V , Regunath H. Hospital acquired infections. In: StatPearls. StatPearls Publishing, 2022. Accessed January 15, 2023. http://www.ncbi.nlm.nih.gov/books/NBK441857/ 28722887

[3] Magill SS , Edwards JR , Bamberg W , et al. Multistate point-prevalence survey of health care-associated infections. N Engl J Med. 2014;370:1198–1208. 24670166 10.1056/NEJMoa1306801 PMC4648343

[4] Bakullari A , Metersky ML , Wang Y , et al. Racial and ethnic disparities in healthcare-associated infections in the United States, 2009-2011. Infect Control Hosp Epidemiol. 2014;35,Suppl 3:S10–S16. 10.1086/677827 25222888

[5] Chen J , Khazanchi R , Bearman G , Marcelin JR. Racial/ethnic inequities in healthcare-associated infections under the shadow of structural racism: narrative review and call to action. Curr Infect Dis Rep. 2021;23:17. 34466126 10.1007/s11908-021-00758-x PMC8390539

[6] Jeon CY , Muennig P , Furuya EY , Cohen B , Nash D , Larson EL. Burden of present-on-admission infections and healthcare-associated infections by race and ethnicity. Am J Infect Control. 2014;42:1296–1302. 25465260 10.1016/j.ajic.2014.08.019 PMC4255287

[7] Gualandi N , Mu Y , Bamberg WM , et al. Racial disparities in invasive methicillin-resistant staphylococcus aureus infections, 2005-2014. Clin Infect Dis Off Publ Infect Dis Soc Am. 2018;67:1175–1181. 10.1093/cid/ciy277 PMC6232852 29659728

[8] Jacobs ZG , Prasad PA , Fang MC , Abe-Jones Y , Kangelaris KN. The association between limited English proficiency and sepsis mortality. J Hosp Med Online. 2020;15:140–146. 10.12788/jhm.3334 PMC7064297 31891556

[9] Karliner LS , Kim SE , Meltzer DO , Auerbach AD. Influence of language barriers on outcomes of hospital care for general medicine inpatients. J Hosp Med. 2010;5:276–282. 20533573 10.1002/jhm.658

[10] Rha B. Vital signs: Health disparities in hemodialysis-associated staphylococcus aureus bloodstream infections — United States, 2017–2020. MMWR Morb Mortal Wkly Rep. 2023;72. 10.15585/mmwr.mm7206e1 PMC9925139 36757874

